# Complete Genome Sequence of Middle East Respiratory Syndrome Coronavirus Isolated from a Dromedary Camel in Egypt

**DOI:** 10.1128/genomeA.00309-16

**Published:** 2016-04-28

**Authors:** Ahmed Kandeil, Mahmoud M. Shehata, Rabeh El Shesheny, Mokhtar R. Gomaa, Mohamed A. Ali, Ghazi Kayali

**Affiliations:** a Center of Scientific Excellence for Influenza Viruses, National Research Centre, Giza, Egypt; b Department of Infectious Diseases, St. Jude Children’s Research Hospital, Memphis, Tennessee, USA; c Department of Epidemiology, Human Genetics, and Environmental Sciences, University of Texas Health Sciences Center, Houston, Texas, USA; d Human Link, Hazmieh, Lebanon

## Abstract

We generated the near-full genome sequence of Middle East respiratory syndrome coronavirus (MERS-CoV) from a collected nasal sample of dromedary camel in Egypt. The newly characterized Egyptian strain has high similarity to the previously characterized Egyptian virus and both of viruses fell into a cluster distinct from other MERS-CoVs.

## GENOME ANNOUNCEMENT

The Middle East respiratory syndrome coronavirus (MERS-CoV) is a new member of the *Betacoronavirus* genus. It was first detected in 2012 in samples of an infected human and was later found to be related to camels (1, 2). MERS-CoV is a positive-sense, single-stranded RNA virus, encoding 11 proteins: two replicase polyproteins (open reading frames [ORFs] 1ab and 1a), four structural proteins (surface spike glycoprotein [S], envelope [E], nucleoprotein [N], and membrane protein [M]), and five nonstructural proteins (ORFs 3, 4a, 4b, 5, and 8b) (2).

The first complete genome of MERS-CoV from an infected dromedary camel (*Camelus dromedarius*) in Egypt was named NRCE-HKU205 and deposited in GenBank under accession number KJ477102 (3). Here, we report the near full genome sequence of another MERS-CoV detected through systematic surveillance in Egypt. It was detected in the nasal swab from a healthy adult dromedary camel collected on 17 December 2014 from an abattoir in Cairo, Egypt.

Viral RNA was extracted using a QIAmp viral RNA minikit (Qiagen, Germany). Reverse transcription was performed using the Superscript III system (Invitrogen, Carlsbad, CA) with random hexamers. The cDNA was subjected to 11 PCRs to generate overlapping amplicons covering the full-length MERS-CoV genome as previously described (4). The PCR products were purified from agarose gels using a QIAquick gel extraction kit (Qiagen). Purified amplicons were Sanger sequenced according to the primer/amplicon combinations (123 sequencing reactions) as shown previously (4) at the Macrogen sequencing facility (Macrogen, South Korea). Finally, 29,949 nucleotides (nt) were assembled using SeqMan (DNASTAR, Madison, WI) with a G+C content of 41.2%.

The NRC163 strain shows the typical MERS-CoV genome order with 5′ untranslated region (UTR) (nt 1 to 199), ORF1ab and ORF1a (nt 200 to 21,508), S (nt 21,377 to 25,438), ORF3 (nt 25,453 to 25,764), ORF4a (nt 25,773 to 26,102), ORF4b (nt 26,014 to 26,754), ORF5 (nt 26,761 to 27,435), E (nt 27,511 to 27,759), M (nt 27,774 to 28,433), N (nt 28,487 to 29,728), ORF8b (nt 28,683 to 29,021), and 3′ UTR (nt 29,729 to 29,949). The closest strains to the NRC163 were NRCE-HKU205 and human betacoronavirus 2c Jordan-N3/2012 (accession no. KC776174.1) with 99.5% similarity.

To further investigate the genetic relationship between Egyptian MERS-CoVs and other strains whose genomes are available in GenBank, we performed whole-genome phylogenetic analysis using MEGA 5 (5). Egyptian strains fell into a cluster distinct from other MERS-CoVs detected elsewhere.

Sequence analysis was performed using BioEdit (6). Egyptian MERS-CoVs had 14 characteristic amino acids (aa) in the ORF1ab protein (Y581, F664, F1024, F1583, T1911, L1970, I2000, V2333, C2481, L2639, T2676, S3361, I3721, M5537). No specific aa variations were identified in the S, ORF3, ORF4a, ORF4b, E, M, and N proteins. An aa deletion that was previously identified at site 1,293 of the S protein of NRCE-HKU205 (3) was not identified in NRC163. The biological impact of such differences among MERS-CoV strains needs to be fully examined.

### Nucleotide sequence accession number.

The complete genome sequence of the MERS-CoV/Egypt/NRC163/2014 was deposited in GenBank under the accession number KU740200.
